# “Extroverted Cuff”, a novel modification of the elephant trunk technique for distal anastomosis of ragged descending aorta

**DOI:** 10.1186/s13019-016-0434-y

**Published:** 2016-04-02

**Authors:** Edvin Prifti, Altin Veshti, Fadil Ademaj, Arben Baboci

**Affiliations:** Division of Cardiac Surgery, University Hospital Center of Tirana, Tirana, Albania; Division of Cardiology, Gjakovo Hospital, Rr. Prizren, Gjakovo, Kosovo

**Keywords:** Elephant trunk technique, Ragged aorta, Aortic dissection

## Abstract

**Background:**

The elephant trunk technique has been applied in various situations including distal aortic dissection, entire aortic replacement, proximal aortic aneurysm, proximal aortic dissection, and Marfan’s syndrome. The elephant trunk technique remains a challenge in cardiac surgery. Here we report a modification of this surgical approach.

**Case presentation:**

The “extroverted cuff” technique that we propose is a novel modification of the flanged technique for the elephant trunk construction. The technique consists in the graft extroversion which is than located inside the descending aorta. Then the distal anastomosis is constructed between the descending aorta and the circular free edge the external layer of the graft. Such a technique was successfully applied in a patient with type A aortic dissection undergoing modified elephant trunk technique, aortic arch replacement and Bentall operation.

**Conclusion:**

Such a modification seems suitable for aortic arch aneurysm with ragged descending thoracic aorta that minimizes bleeding from the distal anastomosis and potentially prevents distal embolization of atheromatous plaque.

## Background

The elephant trunk was introduced by Borst et al. [1] for a staged approach in extensive aortic disease. The elephant trunk has been applied in various situations including distal aortic dissection, entire aortic replacement, proximal aortic aneurysm, proximal aortic dissection, and Marfan's syndrome. Modifications of the elephant trunk have been reported, however the distal anastomosis, especially the posterior aspect, is very difficult to be exposed after been constructed, and bleeding remains an important surgical pitfall [2–5]. We present a novel modification of the elephant trunk indicated for aortic arch aneurysm with ragged descending thoracic aorta that minimizes bleeding from the distal anastomosis and potentially prevents distal embolization of atheromatous plaque.

## Case presentation and surgical technique

**Fig. 1:**
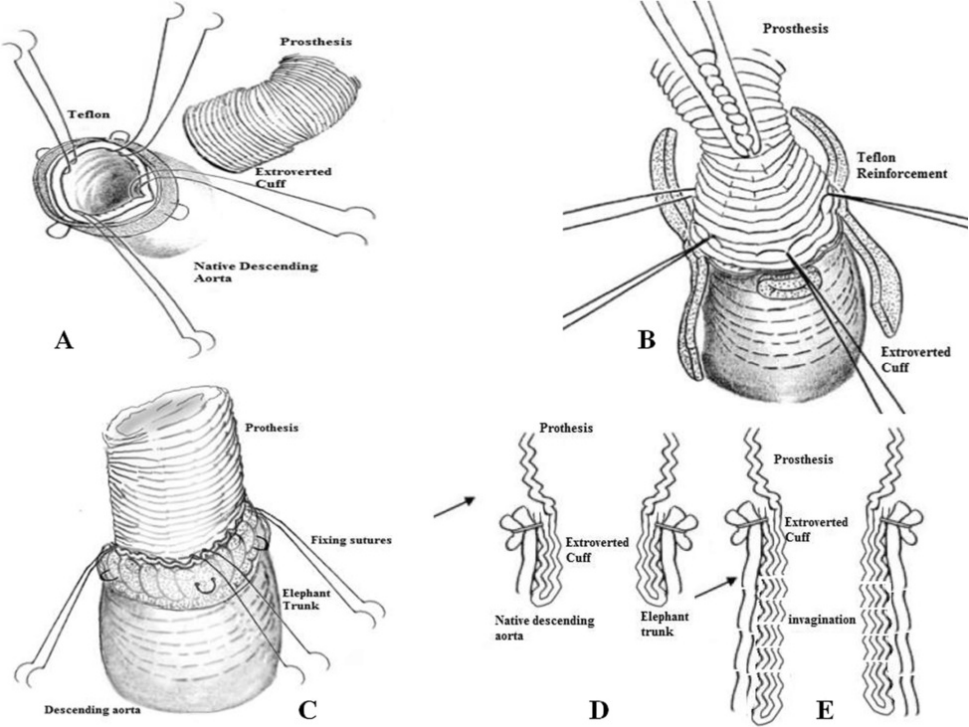
**a** The descending aorta is reinforced with a strip of Teflon and the graft is extroverted. **b** The graft is inserted inside the native descending aorta. **c** Completion of the distal anastomosis between the external layer of the “extroverted cuff” and the native descending aorta with the fixing sutures. **d** Transversal view of the extroverted graft. **e** Further invagination of the “cuff” inside the native descending aorta (represented in dashed lines)

**Fig. 2:**
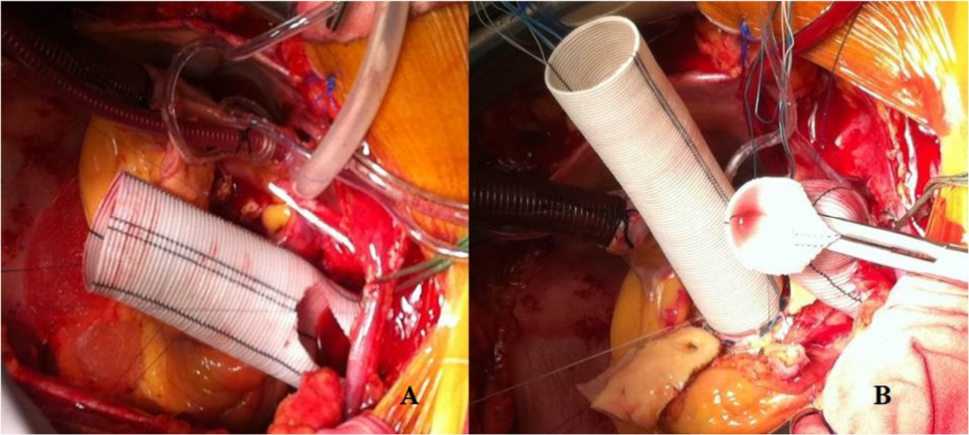
**a** After the distal anastomosis completion, initiation of the epiaortic button implantation. **b** The anastomosis between the graft and the composite graft

**Fig. 3:**
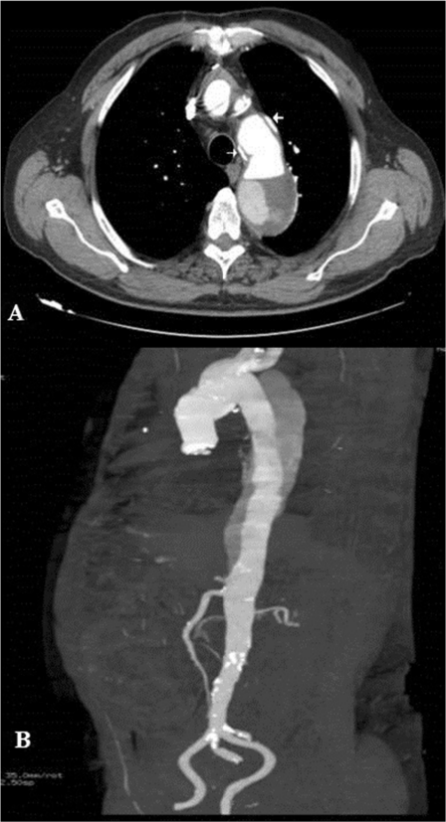
**a** The contrast enhanced angio-CT at one week after surgery demonstrating the initial segment of the “extroverted cuff”. **b** The contrast enhanced angio-CT at two years demonstrating a normally functioning elephant trunk. Legend: The arrows demonstrate the “extroverted cuff”

A 62 years old male presented Type A aortic dissection underwent emergent surgery. Cardiopulmonary bypass was instituted with right atrial drainage and femoral artery cannulation. The ascending aorta was clamped and then opened and carefully inspected, identifying the intimal tear extending into the aortic arch. The patient was cooled to a rectal temperature of 22 °C, and then the circulatory arrest was instituted associated with retrograde cerebral perfusion. The epiaortic vessels were prepared as a single button. The descending aorta was carefully transected and its inner dimensions were examined to select a graft that properly fit and a severely ragged descending aorta wall was diagnosed. Horizontal mattress stitches were placed in the descending thoracic aorta, while applying a Teflon felt strip on the outside of the transected aorta (Fig. 1a). A 30 mm Hemashield (30 cm length) woven double velour, Meadox, USA) graft was selected, the distal part was extroverted as a “cuff” for about 4 cm of the graft which were carefully located inside the descending thoracic aorta (Fig. 1a and 1b). Then the distal anastomosis was constructed between the descending thoracic aorta and the circular free edge of the extroverted graft using a 4/0 continuous prolene suture. After the completion of the distal anastomosis (Fig. 1d), the descending thoracic aorta was inspected and the graft was invaginated futher inside the native descending thoracic aorta with an extra 4–5 cm (Fig. 1e). Then, four horizontal mattress stitches between the reinforced rim of the native aorta and the prosthesis (preferably reinforced with pledgets) were passed from inside the graft to outside including the reversed section of the graft and then were tied down, fixing definitively the length of the graft serving as an elephant trunk (Fig. 1cb). The superior aspect of the graft was prepared to accomodate the button of the epiaortic vessels which were implanted into the graft (Fig. 2a). Then the perfusion was restarted. The distal anastomosis was carefully inspected for any leakage. The distal anastomosis is a flexible structure and the visualization of the posterior aspect of the anastomosis was easier, simply by pressing with a forceps the graft towards the inferior aspect. A Bentall operation consisting in the implantation of a 23 mm Valve tube (St Jude) (Fig. 2b). One week later a contrast enhanced angio-CT demonstrated excellent outcome, however the elephant trunk was not totally attached to the descending thoracic aorta and the constructed “extroverted cuff” was easily identified (Fig. 3a). Two years after surgery an angio-CT demonstrated a normal perfusion of the distal aorta and normally functioning elephant trunk, totally attached to the native descending thoracic aorta (Fig. 3b).

## Discussion

The elephant trunk is employed in distal aortic dissection, entire aortic replacement, proximal aortic aneurysm, proximal aortic dissection, and Marfan’s syndrome [1]. Furthermore, technical modifications such as distal reconstruction using horizontal interrupted buttressed sutures and use of a single four-branched graft with a "collar" and a long "elephant trunk", have been reported [4].

Elephant trunk construction can be performed according the stepwise technique and the flanged graft technique. The stepwise technique includes two methodology such as the graft’s invagination and then pull-out procedure and the second stepwise technique consists in insterting a tube without invagination into the descending thoracic aorta as an elephant trunk. In both stepwise techniques a second graft should be employed to reconstruct the aortic arch. The stepwise technique may be associated with uncontrollable bleeding even after meticulous anastomosis, especially the posterior aspect of the distal anastomosis. This is a troublesome pitfall of the stepwise technique.

The flanged techniques consist in the construction of a circular ridge or flanged and then the distal side of the prothesis is inserted into the trimmed end of the descending aorta in a nearly blind manner and the distal anastomosis is constructed. Despite the distal anastomosis is much more visible in the flanged techniques, the correction of bleeding remains still a pitfall of such technical modifications. The flange techniques require the incorporation of frozen elephant trunks thrombi or atheromatous lesions are recognized. Different associated modification have been reported such as using of a single four-branched graft with a “collar” [4], construction of a circular ridge at the prosthesis [2], flanged graft technique [3], mini elephant trunk [5].

The employement of a stented graft according to various reported hybrid techniques, seems to offer excellent outcome and its implantation is relatively straightforward [6]. However the stented prosthesis costs remain extremely high. Also in ragged descending aorta the employment of a stented graft might be associated with thrombus or atheromatous material dislogment during the implantation.

Our modification seems to be suitable in aortic arch aneurysm with ragged descending thoracic aorta. Such a modification consisting in an “extroverted cuff” created by graft’s invagination, minimizes the bleeding from the distal anastomosis because the continuity of the woven graft is maintained and because the graft is expanded by the blood pressure so as to fit more snugly to the aortic wall. Also the distal anastomis is much more visible and the graft much more flexible permitting a better visualization of the posterior aspect of the distal anastomosis. Differently to another technique reported by Kanagasabay et al [4], our modification offers the opportunity of constructing the elephant trunk without inserting the entire length of the invaginated graft inside the descending thoracic aorta, which might detach atheromatous plaques during the distal anastomosis construction. Once the distal anastomosis is constructed the length of the elephant trunk is decided and the invaginated segment of the graft is increased by pushing the graft inside the descending thoracic aorta and then fixed with separated stitches.

Such a modification has the advantage of preventing distal embolization of atheromatous plaque by creating an adequate and effective contact area between the graft and ragged aortic wall. Such a technique does not require a second graft employment. Also, the elephant trunk, which consists in the invaginated segment of the graft, is composed by a double layer graft attached to the native descending thoracic aorta, and the entire length of the elephant trunk is much more sustained, and the radial force exerted by the elephant trunk to the fragile aortic wall is much more uniform, avoiding the possible distortion of the graft, promoting thrombosis of the false lumen, and contributing to the shrinkage of the aorta as demonstrated in our case. Furthermore, the “extroverted cuff” itself enables the placement of certain everted sutures between the graft and the trimmed descending thoracic aorta, resulting in a stable and secure suture line. It would also be easier to apply additional everted sutures to control bleeding.

## Conclusions

As conclusion, we believe that the “extroverted cuff” technique is a new modification, enabling an easy construction of the elephant trunk, especially in ragged aorta, and a better exposing of the posterior aspect of the anastomosis, permitting an easy correction of possible bleeding which should be part of the surgical armamentarium.

## Statement

Ethics approval was received by the institutional ethics committee. The 'Consent to publish' was received by the patient and the family.
